# Laparoscopic Sleeve Gastrectomy for an Obese Patient with Retroperitoneal Schwannoma: A Case Report

**DOI:** 10.70352/scrj.cr.25-0419

**Published:** 2025-10-09

**Authors:** Yuriko Yamada, Shin Saito, Shinichiro Yokota, Yoshinori Hosoya, Hiroharu Yamashita, Hironori Yamaguchi

**Affiliations:** 1 Department of Surgery, Jichi Medical University, Shimotsuke, Tochigi, Japan; 2 Division of Abdominal Transplant, Department of Surgery, Stanford University School of Medicine, Stanford, CA, USA

**Keywords:** laparoscopic sleeve gastrectomy, retroperitoneal schwannoma, intraoperative frozen section, morbid obesity

## Abstract

**INTRODUCTION:**

The prevalence of obesity has increased markedly across the globe over the past several decades. In parallel, the number of metabolic surgeries has risen sharply in recent years, resulting in a growing incidence of concomitant surgical procedures. Obese individuals have an elevated risk of malignancy, partly due to the chronic inflammation associated with excess adipose tissue. A key clinical question arises: How should surgeons manage unexpected pathology identified during the preoperative evaluation of patients with morbid obesity?

**CASE PRESENTATION:**

A morbidly obese male patient with a body mass index of 50 underwent evaluation for laparoscopic sleeve gastrectomy (LSG). Preoperative CT and PET-CT revealed a 25-mm retroperitoneal mass adjacent to the left gastric artery. Given the elevated risk of malignancy in obese individuals, it was essential to rule out a malignant process before proceeding with LSG. Thus, we adopted a surgical strategy in which LSG would be performed only if the intraoperative frozen section confirmed the lesion to be benign. The retroperitoneal mass was resected laparoscopically and identified as a benign schwannoma on the intraoperative frozen section, allowing us to proceed with LSG as planned. Retroperitoneal schwannomas are rare and often difficult to diagnose preoperatively, which supports the rationale behind our surgical strategy. To our knowledge, this is the first reported case of concomitant LSG performed following the laparoscopic complete excision of a retroperitoneal schwannoma.

**CONCLUSIONS:**

Concomitant procedures performed alongside metabolic surgery may be a viable approach to reduce the overall surgical burden on patients. Given the increased risk of malignancy in morbidly obese individuals, a thorough preoperative evaluation for concomitant malignancies should be considered before bariatric surgery. When an unexpected abdominal pathology is identified on preoperative imaging in obese patients, the intraoperative frozen section analysis of the resected specimen can be a valuable tool to guide surgical decision-making regarding concomitant bariatric surgery.

## Abbreviations

BMI
: body mass index
CD34
: cluster of differentiation 34
GIST
: gastrointestinal stromal tumor
LSG
: laparoscopic sleeve gastrectomy

## INTRODUCTION

The incidence of obesity has escalated markedly across the globe in recent years.^1)^ As a result, metabolic disorders linked to obesity have emerged as major public health challenges worldwide.^2)^ These disorders extend beyond metabolic diseases to include malignancies, with obesity accounting for an estimated 11.9% of cancers in men and 13.1% in women.^3)^ Thus, when unexpected pathology is identified during preoperative examinations of morbidly obese patients, physicians should consider the possibility of underlying malignancy.

LSG is an established surgical procedure for the treatment of morbid obesity.^4)^ Concomitant surgeries with LSG commonly include cholecystectomy^5)^ and resection for GIST; however, resection for gastric schwannoma and leiomyoma are reported only rarely.^6,7)^ One-stage laparoscopic adrenalectomy combined with LSG has been reported previously.^5,8)^ In morbidly obese patients diagnosed with early-stage or low-risk tumors that do not require immediate intervention, LSG may be considered as a bridging strategy before definitive oncologic resection.^9)^ However, bariatric surgery is generally contraindicated in obese patients with advanced malignancy, as they may require systemic treatment such as chemotherapy or are at risk for developing metastasis or recurrence.

A previous report noted that retroperitoneal schwannomas are rare tumors that are challenging to diagnose preoperatively.^10)^ We report a case of a patient who underwent simultaneous LSG and retroperitoneal schwannoma resection, with intraoperative frozen section guiding intraoperative management.

## CASE PRESENTATION

**Fig. 1:**
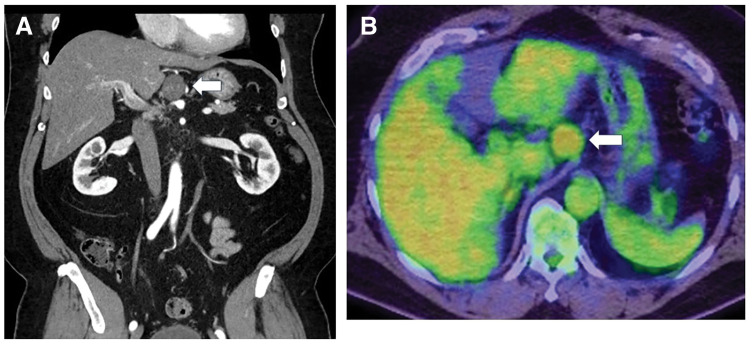
(**A**) An abdominal dynamic contrast-enhanced CT scan showed a 25-mm well-defined, hypodense tumor (arrow) adjacent to the left gastric artery. (**B**) PET-CT detected the tumor with an SUVmax of 2.3 (arrow), without evidence of distant metastases. SUVmax, maximum standardized uptake value

**Fig. 2:**
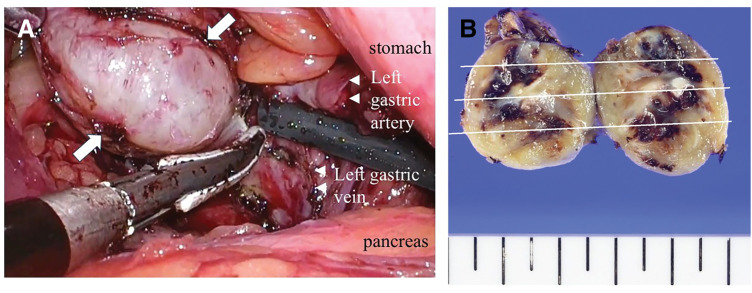
(**A**) The tumor was located behind the left gastric vein, adjacent to the left gastric artery (arrowhead). It was enucleated laparoscopically (arrow). (**B**) Macroscopically, the cut surface of the resected specimen showed an encapsulated, well-circumscribed tumor measuring 25 mm in diameter.

**Fig. 3:**
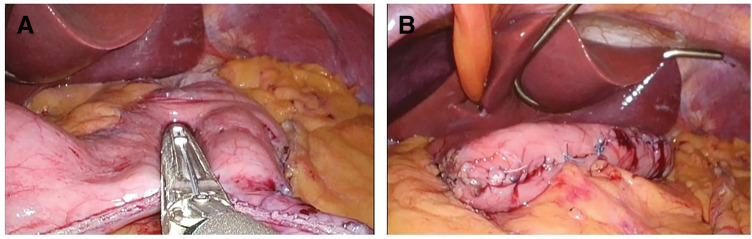
(**A**) Gastric resection was performed with an automatic anastomosis device. The residual stomach is on the left side of the image. (**B**) Staple line reinforcement was added using nonabsorbable imbricated running sutures.

A 55-year-old Japanese male with a history of type 2 diabetes mellitus, hyperlipidemia, hypertension, obstructive sleep apnea, severe hepatic steatosis, and morbid obesity (BMI 50) was referred to our department for surgical evaluation. He underwent preoperative surgical evaluation for LSG. Preoperative imaging, including contrast-enhanced CT and PET-CT, revealed a 25-mm retroperitoneal tumor adjacent to the left gastric artery (**Fig. 1A** and **1B**). Based on imaging characteristics, malignant lymphoma or metastatic lymph node lesions were considered as differential diagnoses. Upper gastrointestinal endoscopy and colonoscopy revealed no evidence of a primary neoplastic lesion. Given the elevated risk for malignancy in obese patients, it was essential to exclude malignancy before proceeding with LSG for this patient. Thus, we adopted a surgical strategy in which LSG would be performed only if the intraoperative frozen section confirmed the lesion to be benign. First, we performed laparoscopic excision of the retroperitoneal mass (**Fig. 2A**), which revealed an encapsulated, round, white, solid tumor (**Fig. 2B**). Intraoperative frozen section analysis suggested that the mass was most likely a benign schwannoma. Although it did not provide a definitive pathological diagnosis of the mass, it did suggest the absence of malignant features. Therefore, LSG was performed as planned (**Fig. 3A** and **3B**). The greater curvature of the stomach was resected using a 37-Fr MIDSLEEVE orogastric calibration tube (MID, Lyon, France) and the Signia Stapling System (Medtronic, Minneapolis, MN, USA) (**Fig. 3A**). Staple line reinforcement was performed using nonabsorbable imbricated running sutures to prevent bleeding and leakage, resulting in a tubular stomach with an approximate volume of 350 mL (**Fig. 3B**). The patient had an uneventful postoperative course without complications.

**Fig. 4:**
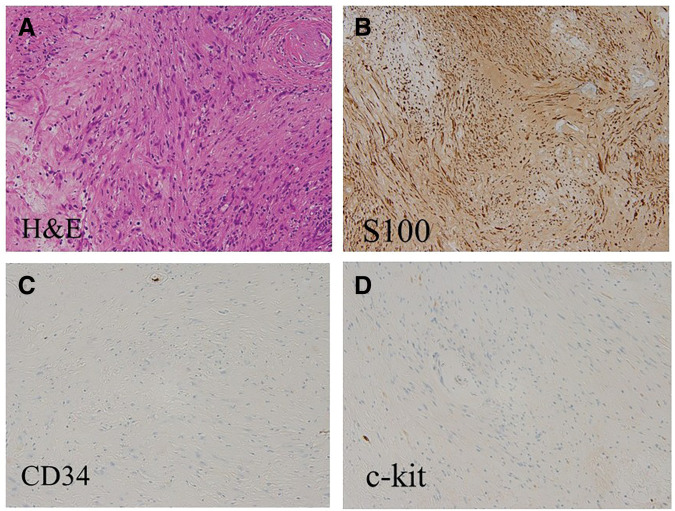
Histologically, the tumor consists of spindle-shaped cells with mild atypia, arranged in whorls and a twisted configuration. (**A**) H&E staining (×200). Immunohistochemistry showed that the tumor cells were positive for S100 protein (**B**) (×200), and negative for CD34 (**C**) (×200) and c-kit (**D**) (×100). The diagnosis of retroperitoneal schwannoma was established based on these findings. CD34, cluster of differentiation 34; H&E, hematoxylin and eosin

Postoperatively, histological examination revealed that the resected specimen showed spindle-shaped cells with mild atypia, arranged in whorls and a fascicular or twisted configuration (**Fig. 4A**). Immunohistochemical study showed strong S-100 positivity (**Fig. 4B**) with a low MIB1 index and negative staining for CD34 and c-kit (**Fig. 4C** and **4D**), suggesting that the tumor was benign. Taken together, the pathological findings were consistent with the diagnosis of benign schwannoma.

Two years after LSG, the patient’s BMI decreased from 50 to 33, with resolution of obesity-related comorbidities. His systolic blood pressure normalized to 130 mmHg without the need for antihypertensive medications, and his hemoglobin A1c improved from 6.1% to 5.9% without medications. Written informed consent was obtained from the patient, and this case report has been prepared in accordance with the SCARE Criteria.^11)^

## DISCUSSION

The prevalence of obesity has increased significantly worldwide over the past several decades and is now considered a global pandemic.^12)^ Consequently, metabolic disorders associated with morbid obesity—such as type 2 diabetes, hypertension, hyperlipidemia, obstructive sleep apnea, coronary artery disease, and nonalcoholic fatty liver disease—have become significant public health concerns worldwide.^2)^ Obesity results from the accumulation of excess adipose tissue, a dynamic endocrine organ that plays a key role in promoting chronic inflammation.^13)^ Obesity promotes tumor development both locally, through inflammation in adipose tissue, and systemically, via inflammatory mediators released as a result of this inflammation.^13)^ Epidemiologic data have established an association between chronic inflammation and the development and progression of several cancers.^14)^ There is strong evidence that excess body weight is associated with an increased risk for cancer at least 13 anatomical sites, including endometrial, esophageal, renal, pancreatic, hepatocellular carcinoma, gastric cardia cancer, meningioma, multiple myeloma, colorectal, postmenopausal breast, ovarian, gallbladder, and thyroid cancers.^3)^

Bariatric surgery is the most effective and durable treatment for morbidly obese patients.^15)^ Among adults with obesity, bariatric surgery compared with nonsurgical intervention was associated with a significantly lower incidence of obesity-associated cancer and cancer-related mortality.^16)^ LSG has rapidly gained popularity due to its technical simplicity and acceptable postoperative outcomes.^2)^ This procedure is associated with 2 factors leading to improvement in obesity-related complications following weight reduction. First, it creates a restrictive limit, causing early satiety by reducing gastric capacity. Second, it eliminates the gastric fundus, which reduces the level of ghrelin, an appetite-stimulating hormone.^17)^

The number of metabolic surgeries has increased dramatically in recent years,^18)^ thereby raising the likelihood of performing concomitant procedures.^5)^ Concomitant surgery is an appealing option, as it reduces the surgical burden on patients requiring treatment for multiple lesions. Previous reports described that 17 submucosal tumors were incidentally identified (1.2%) out of 1415 LSG procedures, including 12 GISTs, 2 schwannomas, and 3 leiomyomas.^7)^ The majority of concomitant procedures with LSG are cholecystectomy^5)^ and resection for GIST.^6)^ Simultaneous laparoscopic adrenalectomy with LSG was also reported previously.^5,8)^

Almiron da R Soares et al. reported in their meta-analysis that patients undergoing concomitant bariatric surgery with cholecystectomy experienced increased rates of postoperative bleeding, wound complications, respiratory complications, and anastomotic complications.^19)^ They concluded that concomitant cholecystectomy should be reserved for high-risk or symptomatic patients and avoided in bariatric procedures involving an anastomosis because of the increased risk of complications.^19)^ Future studies are needed to clarify the effectiveness of 1- versus 2-step operative strategies for morbidly obese patients undergoing bariatric surgery.

LSG can serve as a bridging treatment to definitive oncologic resection in morbidly obese patients with early-stage or low-grade malignancy that does not require immediate intervention.^9)^ The rationale behind this surgical strategy is to reduce surgical complications, as the amount of visceral fat is associated with longer operative time and an increased risk of complications such as surgical site infection and anastomotic failure.^20)^

When preoperative imaging detects unexpected pathology, it may necessitate changes to the surgical plan. Simultaneous LSG and oncologic resection for advanced malignancy is generally not recommended, given the potential need for chemotherapy and the risk of recurrence or metastasis. In morbidly obese patients who are scheduled for bariatric surgery, it is essential to rule out concurrent advanced malignancies.

Schwannomas predominantly occur in the central nervous system or are associated with spinal nerve roots, commonly presenting in the head and neck, upper and lower extremities, posterior mediastinum, and retroperitoneum.^21)^ Retroperitoneal schwannomas are rare tumors that are difficult to diagnose preoperatively.^10)^ In the present case, preoperative imaging incidentally revealed a mass adjacent to the left gastric artery in a morbidly obese patient. We adopted a strategy in which LSG would be performed only if the lesion was diagnosed as benign by intraoperative frozen section analysis. The resected specimen demonstrated a spindle cell tumor with mild atypia on the frozen section, consistent with benign schwannoma. Therefore, LSG was performed as planned.

Although routine bariatric procedures may not necessitate intraoperative pathological assessment, encountering unexpected masses should prompt a multidisciplinary decision-making process involving pathologists, as demonstrated in our case using the intraoperative frozen section analysis.

In hospitals with limited access to rapid intraoperative pathological analysis, we recommend 2 practical approaches for performing bariatric surgery in the presence of an unexpected mass. The 1st approach is a 2-stage procedure, in which the mass is resected first, followed by confirmation of the pathological diagnosis before proceeding with bariatric surgery, even though this requires the patient to undergo 2 separate operations. The 2nd approach is referral to a high-volume center. Because the safety of a 1-stage surgical plan depends on rapid intraoperative pathologic evaluation, patients with suspicious findings should be considered for transfer to a high-volume center capable of providing intraoperative diagnosis.

Intraoperative frozen section analysis has limitations. It may have limited ability to distinguish low-grade malignancy from benign spindle cell tumors, which typically require immunohistochemical study for definitive diagnosis. There is also a risk of sampling error; therefore, intraoperative frozen section analysis cannot definitively rule out malignancies in retroperitoneal masses.

To the best of our knowledge, we are reporting the first case of simultaneous LSG with laparoscopic resection of retroperitoneal schwannoma using intraoperative frozen section analysis. We selected LSG for this patient with retroperitoneal schwannoma because this procedure can be simpler and associated with fewer complications, although other bariatric surgeries may offer superior outcomes in terms of weight loss and metabolic comorbidity reduction.^2,4,15,17)^

## CONCLUSIONS

Morbidly obese patients are at an increased risk of malignancy. When an incidental abdominal tumor is identified on preoperative imaging, intraoperative frozen section analysis can play a crucial role in determining whether concomitant resection should be performed alongside bariatric surgery. Simultaneous surgery can be cost-effective and appealing to patients; however, clear communication regarding the risks and benefits of simultaneous procedures is essential. Additionally, such surgery requires a well-trained, specialized surgeon, as well as a reliable anesthesia and pathology team.
